# Rapamycin attenuated podocyte apoptosis via upregulation of nestin in Ang II-induced podocyte injury

**DOI:** 10.1007/s11033-021-07029-x

**Published:** 2022-02-11

**Authors:** Huimin Shi, Yajie Zhao, Tiantian He, Xianli Wen, Gaoting Qu, Shanwen Li, Weihua Gan, Aiqing Zhang

**Affiliations:** Department of Pediatric Nephrology, The Second Affiliated Hospital of Nanjing Medical University, 262 Zhongshan North Road, Nanjing, 210003 Jiangsu Province China

**Keywords:** Nestin, Rapamycin, Ang II, Podocyte injury, Apoptosis

## Abstract

**Background:**

Angiotensin II (Ang II) contributes to the progression of glomerulosclerosis, mainly by inducing podocyte injury. Convincing evidence indicates that the mTOR inhibitor rapamycin could play a fundamental role in protection against podocyte injury. Nestin, a major cytoskeletal protein, is stably expressed in podocytes and correlates with podocyte damage. The purpose of this study was to investigate the effect of rapamycin on podocyte injury induced by Ang II and to clarify the role and mechanism of nestin in the protective effect of rapamycin of podocyte injury.

**Methods and results:**

We established an Ang II perfusion animal model, and the effects of rapamycin treatment on podocytes were investigated in vivo. In vitro, podocytes were stimulated with Ang II and rapamycin to observe podocyte injury, and nestin-siRNA was transfected to investigate the underlying mechanisms. We observed that Ang II induced podocyte injury both in vivo and in vitro, whereas rapamycin treatment relieved Ang II-induced podocyte injury. We further found that nestin co-localized with p-mTOR in glomeruli, and the protective effect of rapamycin was reduced by nestin-siRNA in podocytes. Moreover, co-IP indicated the interaction between nestin and p-mTOR, and nestin could affect podocyte injury via the mTOR/P70S6K signaling pathway.

**Conclusion:**

We demonstrated that rapamycin attenuated podocyte apoptosis via upregulation of nestin expression through the mTOR/P70S6K signaling pathway in an Ang II-induced podocyte injury.

## Introduction

Podocytes, which maintain the integrity of the glomerular filtration barrier, are terminally highly differentiated in the renal glomerulus [1]. Podocyte injury causes proteinuria, leading to the progression of glomerular diseases [2]. Angiotensin II (Ang II), an important active effector in the renin-angiotensin system (RAS), contributes to podocyte injury, including podocyte apoptosis, cytoskeletal rearrangement, and loss of the slit diaphragm [3–5]. Several prior studies have shown that podocyte injury is closely related to the activation of the mammalian target of rapamycin (mTOR) [6–9], which plays an important role in glomerular disease. Rapamycin, an mTOR inhibitor, has been shown to have immunosuppressive and antiproliferative efficiency [10, 11]. It has been revealed that rapamycin treatment protected a rat model of diabetic nephropathy from kidney impairment [12].

Nestin is a class VI intermediate filament (IF) protein that was initially characterized in neuroepithelial stem cells [13] and participates in the composition of the cytoskeleton. Nestin is stably expressed in mature podocytes and plays a significant role in maintaining the normal morphology and function of podocytes [14, 15]. However, the signaling mechanism involved in nestin function in glomerular podocytes has not yet been established.

We hypothesized that rapamycin attenuates podocyte injury under Ang II conditions and that nestin could play an important role in this process. Therefore, in this study, we explored the effects of rapamycin on podocytes by investigating the changes in nestin expression and elucidating the underlying mechanisms.

## Materials and methods

### Animals

Eight-week-old, 20–22-g male C57BL/6 mice (Vital River Laboratory Animal Technology, Beijing, China) were included in the experiments. The experimental procedures were approved by the Institutional Animal Care and Use Committee of the Nanjing Medical University. The mice underwent a sham operation under light anesthesia with 3% isoflurane. Two weeks after surgical intervention, an osmotic minipump (model 2004; Alzet, Cupertino, CA) was implanted subcutaneously in mice to infuse Ang II for 4 weeks. The mice were randomly divided into three experimental groups: control group (sham operation, n = 6); Ang II group (Ang II, 400 ng/kg/min [16], subcutaneously-embedded anosmotic minipump, n = 6); and Ang II + RP group (Ang II, 400 ng/kg/min, subcutaneously-embedded minipump, intraperitoneal injection 1 mg/kg/day RP, n = 6). The drinking water of all groups was supplemented with 1% sodium chloride throughout the experimental period. Kidneys were collected for biochemical and renal pathological analyses.

### Cell culture

The immortalized mouse podocyte cell lines (MPCs) used in this study were gifted by Dr. Junwei Yang (Nanjing Medical University). Podocytes were cultured in RPMI-1640 medium (Gibco, USA) with 10% fetal bovine serum (FBS), 100 U/ml penicillin G (Gibco, USA), and 10 U/ml recombinant mouse IFN-γ at 33 °C for proliferation. Podocytes were cultured without IFN-γ at 37 °C for 10–14 days to induce differentiation and quiescence. The cells were then exposed to Ang II (10^–6^ mol/l) and rapamycin (500 ng/ml) treatment for the indicated time periods.

### Transfection of small interference RNA

MPCs were transfected with nestin siRNA (sense, 5′- GGAAGUGACUAGUGAGACATT-3′ and antisense, 5′- UGUCUCACUAGUCACUUCCTT-3′) (GeneChem, Shanghai, China) using Lipofectamine 2000 transfection reagent (Invitrogen, USA), according to the manufacturer’s instructions. In brief, nestin siRNA was diluted into each 6-well plate at a concentration of 40 nM with transfection medium (Opti-MEM, Invitrogen, USA) and incubated for 5 min. Diluted lipofectamine reagent with Opti-MEM and siRNA was mixed and incubated at room temperature for 20 min. After 6 h of transfection, the cells were used for further experiments.

### Immunohistochemistry

Immunohistochemical staining was performed on 4% paraformaldehyde-fixed, paraffin-embedded 3-μm renal tissue sections. After antigen recovery, the sections were incubated with primary antibodies against nephrin (1:200, mouse, sc-377246) overnight at 4 °C, and then incubated with peroxidase-conjugated anti-mouse IgG antibodies. Reactions were stained with a DAB substrate kit (MXB biotechnologies, DAB-0031, Fuzhou, China), and counterstaining was performed using hematoxylin. The sections were captured under a microscope (Olympus, BX53, Tokyo, Japan), and Image ProPlus was used to quantify the average optical density value.

### Immunofluorescence staining

Kidney paraffin sections (3 μm thick) were prepared. Cells grown on glass cover slips were fixed with 4% paraformaldehyde for 15 min and permeabilized with 0.1% Triton X-100 for 5 min at room temperature. After blocking with 5% BSA for 1 h, the kidney and cell slides were incubated with primary antibodies against WT1(1:200, rabbit, #83,535, Cell Signaling Technology), p-mTOR (1:100, rabbit, #5536, Cell Signaling Technology), nestin (1:200, mouse, sc-23927, Santa Cruz Biotechnology), and nephrin (1:200, mouse, sc-376522, Santa Cruz Biotechnology) in PBS containing 1% BSA at 4 °C overnight. FITC/TRITC-conjugated IgG was used as a secondary antibody for 1 h at room temperature. All samples were mounted with DAPI dye for 5 min and captured under a confocal microscope (Olympus FV1000, Tokyo, Japan).

### Annexin V staining assay

Podocytes from different groups were quantified by annexin V staining according to the manufacturer’s instructions (KeyGEN Biotech, Jiangsu, China). Podocytes were harvested and washed twice with phosphate-buffered saline (PBS). The cells were resuspended in 100 μl of ice-cold binding buffer and incubated with 5 μl of Annexin V (conjugated with FITC) for 15 min in the dark. After resuspension in 400 μl of binding buffer, the cells were observed under a fluorescence microscope (Olympus, BX53, Tokyo, Japan).

### TUNEL assay

Tissue sections (3 μm) were used to detect DNA fragmentation for apoptosis using the One Step TUNEL Apoptosis Assay Kit (KeyGEN Biotech, Jiangsu, China) according to the manufacturer’s instructions. Kidney cells with TRITC nuclear markers were considered TUNEL-positive. The cells were counterstained with DAPI, and fluorescent images were acquired using a confocal microscope.

### Western blotting assay

Renal tissues and cultured cells were harvested after treatment and lysed in RIPA lysis buffer, supplemented with a protease inhibitor cocktail. The BCA assay (KeyGEN Biotech, Jiangsu, China) was used to measure protein concentration. Protein expression was detected by western blotting, according to established protocols [17]. The same quantities of protein were separated by 10% SDS-PAGE and transferred to a PVDF membrane (Millipore, HATF09025). The membranes were blocked with 5% BSA in TBST for 1 h. Membranes were incubated overnight at 4 °C with the following primary antibodies: nephrin (1:1000, mouse, sc-376522, Santa Cruz Biotechnology), Nestin (1:500, mouse, sc-23927, Santa Cruz Biotechnology), Bax (1:1000, rabbit, AF0120, Affinity Biosciences), p53 (1:1000, mouse, BF8013, Affinity Biosciences), and GAPDH (1:1000, rabbit, #5174, Cell Signaling Technology) were used. Secondary HRP-conjugated goat anti-mouse or anti-rabbit antibodies (Cell Signaling Technology) were used. Western ECL substrate (Biosharp, China) was used to visualize proteins.

### CO-immunoprecipitation (CO-IP) and immunoblotting

Cells were lysed in lysis buffer, and proteins were immunoprecipitated from the cell lysates with the indicated primary antibodies overnight at 4 °C, according to the manufacturer’s instructions (abs955, Absin, Shanghai, China). Immunoprecipitates were mixed with protein G agarose beads for 1 h at 4 °C, collected, and washed with lysis buffer. After incubation, the immunoprecipitate complexes were rinsed, and the contents of the sample were analyzed by immunoblotting.

### Statistical analysis

The results are expressed as the mean ± SEM from at least three independent experiments. Statistical analysis was performed using Prism 6.0 for Windows (GraphPad Software, Inc., California, USA). Inter-group comparisons were assessed using ordinary ANOVA followed by Bonferroni’s multiple comparison post-hoc test. A two-sided P-value of < 0.05 indicated statistical significance.

## Results

### Rapamycin attenuated podocyte injury in Ang II-infused mice

**Fig. 1:**
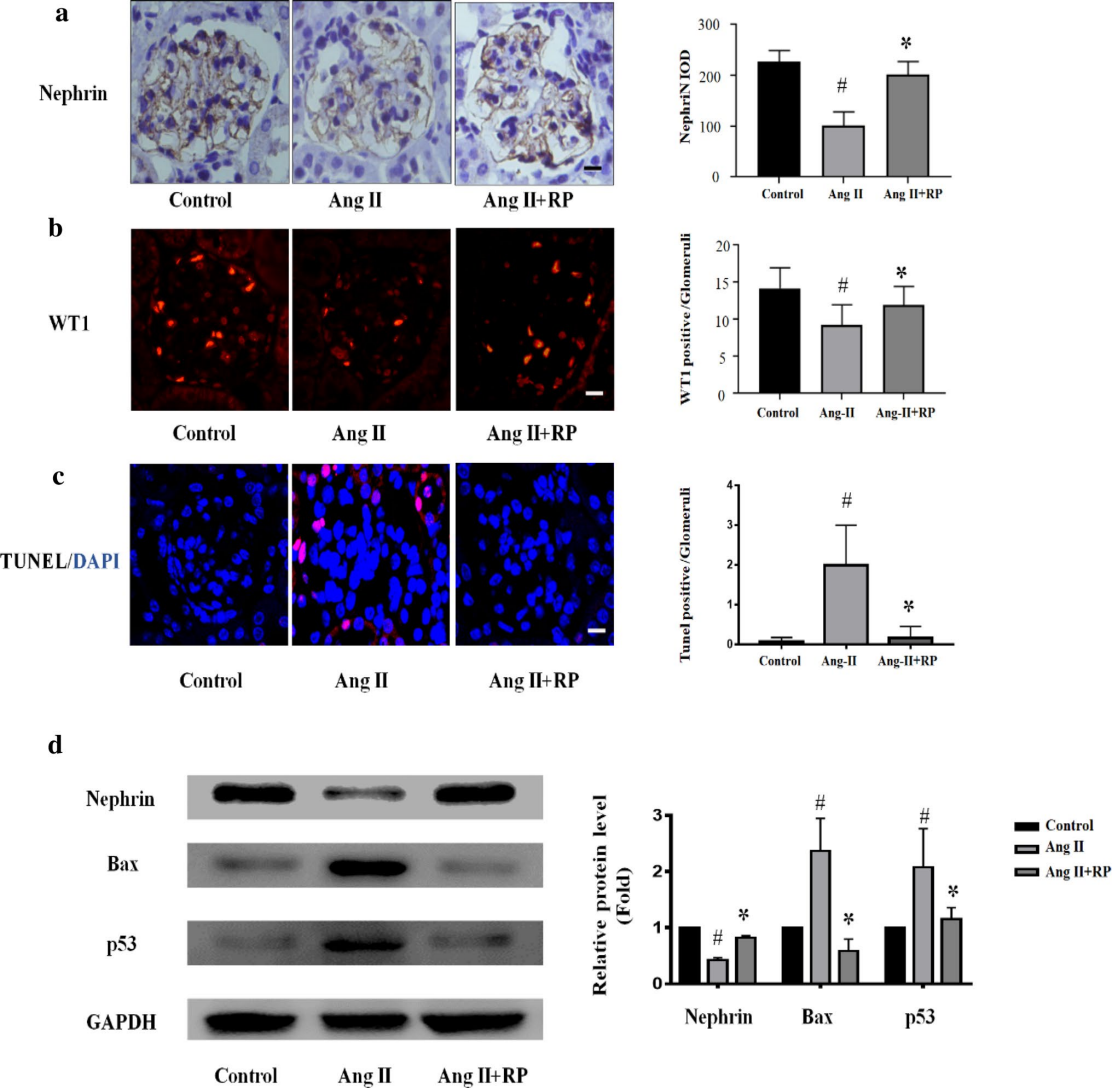
Rapamycin attenuated podocyte injury in Ang II-infused mice. **a** As indicated, Immunohistochemical staining for nephrin in kidney tissue from each group. Magnification, × 400. **b** Representative images of WT-1 immunofluorescent staining in kidney tissues from the above group. Magnification, × 400. **c** TUNEL staining (Red) in kidney sections after the indicated treatment. The sections were counterstained with DAPI (Blue). Magnification, × 400. **d** Western blotting analysis of nephrin, Bax and p53 expression in the kidney. The levels of GAPDH were used as standard loading controls. The data are presented as mean ± SEM. #P < 0.05 vs. control group, *P < 0.05 vs. Ang II group. (Color figure online)

We established an Ang II-infused mouse model to investigate the effects of rapamycin on glomerular and podocyte injury. The dysregulation or loss of nephrin, an important structural molecule in the silt diaphragm, has been suggested to precede podocyte loss. As shown in Fig. 1, the deletion of nephrin was confirmed via immunohistochemistry and western blotting assays of kidney sections after Ang II stimulation compared with that in the control group. In the presence of rapamycin, podocyte injury induced by Ang II was attenuated. A higher expression level of nephrin was observed after injection of rapamycin compared to that in the Ang II group (Fig. 1a and d). Additionally, immunochemical staining for Wilms’ tumor protein (WT1), a surrogate marker of podocyte number, suggested that the number of podocytes was decreased in the Ang II group compared with that in the control group. The number of WT-1 immunopositive podocytes was higher in the glomeruli of the rapamycin-treated group than in glomeruli of the Ang II group (Fig. 1b). The TUNEL assay showed that Ang II induced glomerular apoptosis, and rapamycin treatment could reduce apoptosis (Fig. 1c). The expression of renal apoptosis-related proteins, including Bax and p53, was measured to investigate the effects of rapamycin on renal apoptosis. In the Ang II group, remarkable upregulation of Bax and p53 expression was observed, whereas significant inhibition of Bax and p53 expression was observed after rapamycin treatment (Fig. 1d). These results suggest that rapamycin attenuates podocyte injury in Ang II-infused mice.

### Inhibition of mTOR signaling activation by rapamycin restored nestin expression in the glomeruli of Ang II-infused mice

**Fig. 2:**
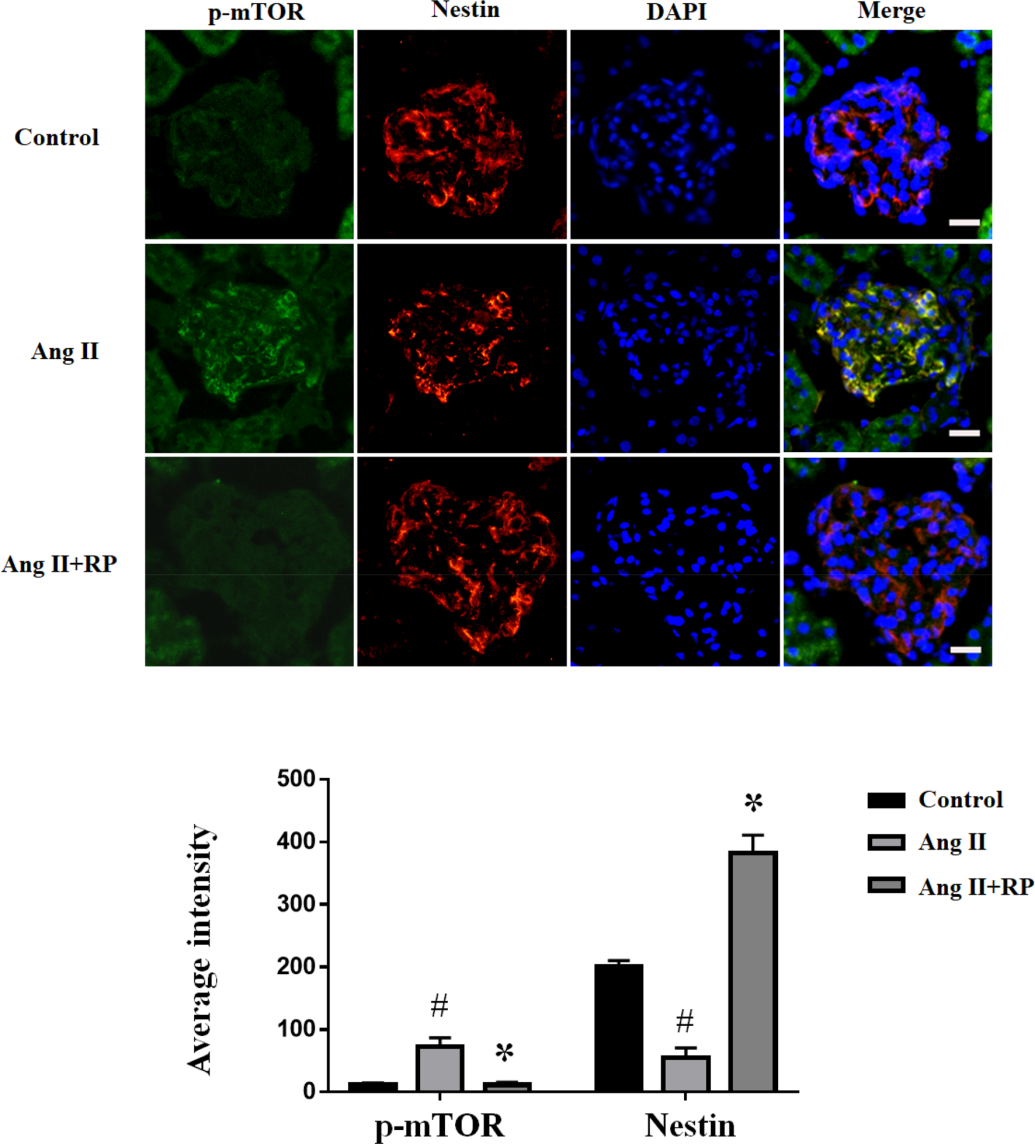
Inhibition of mTOR signaling activation by rapamycin restored nestin expression in glomeruli of Ang II-infused mice. Indirect immunofluorescent staining showed the expression and localization of p-mTOR (green), nestin (red), and DAPI (blue) in glomeruli from various group, as indicated. Magnification, × 400. Fluorescence average intensities of p-mTOR and nestin in glomeruli after the indicated treatment. The data are presented as mean ± SEM. #P < 0.05 vs. control group, *P < 0.05 vs. Ang II group. (Color figure online)

We evaluated the expression level of signal transduction in renal tissue samples using p-mTOR immunofluorescence. As demonstrated in Fig. 2, the expression of p-mTOR in glomeruli was increased significantly, suggesting activation of mTOR signaling. Further, the Ang II + RP group exhibited lower p-mTOR expression than Ang II group. Interestingly, nestin, a cytoskeleton disruption, was co-localized with p-mTOR in the glomeruli. Nestin expression was weakly observed in the glomeruli of the Ang II group. In the rapamycin-treated group, nestin expression was higher. These results indicated that rapamycin attenuated mTOR signaling activation by restoring nestin expression in the glomeruli of Ang II-infused mice.

### Inhibition of nestin attenuated the protective effect of rapamycin against Ang II-induced podocyte injury

**Fig. 3:**
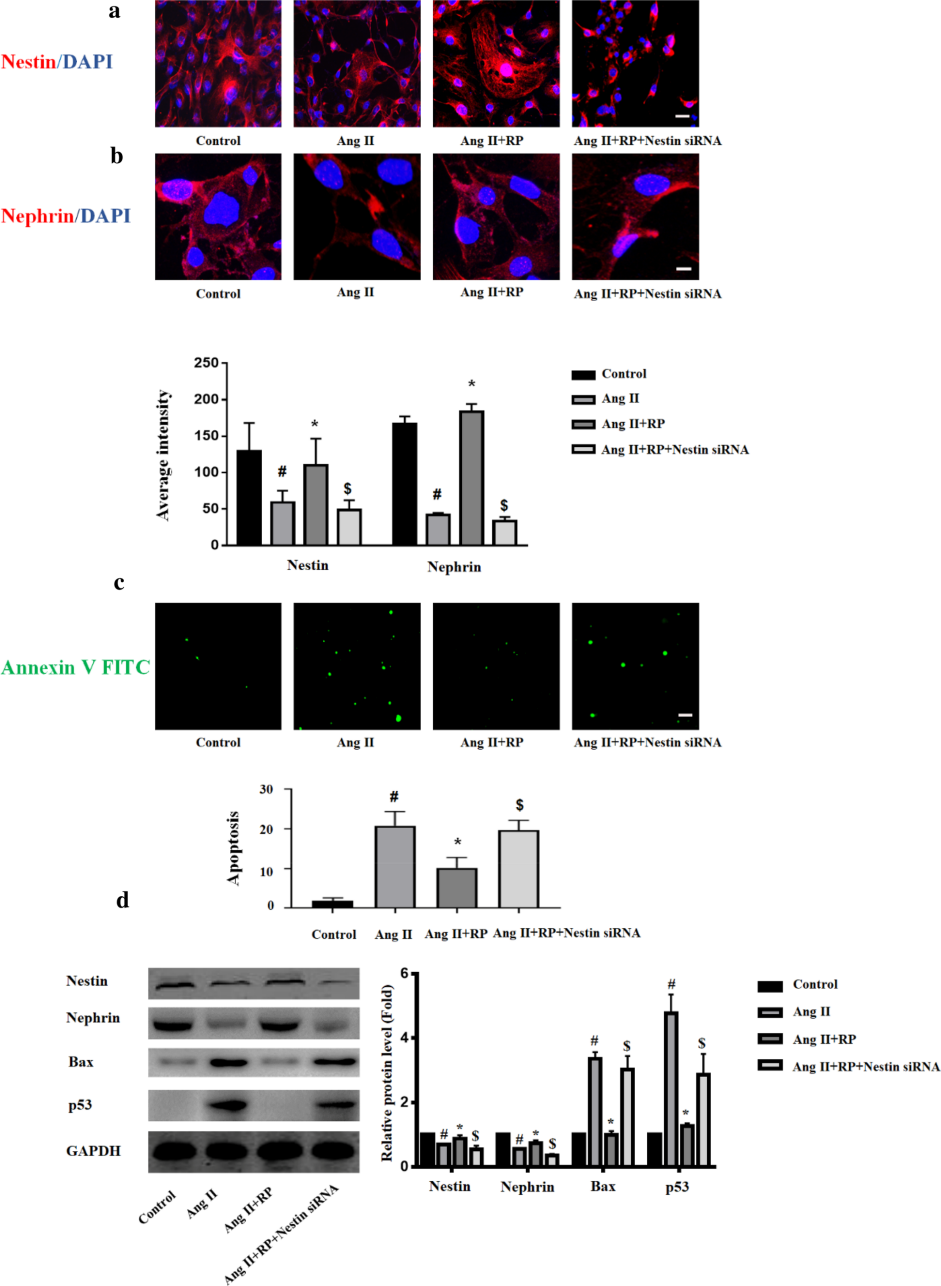
Rapamycin in Ang II-induced podocyte injury. **a** Immunofluorescence staining showed the expression of nestin (Red) in podocyte after the indicated treatment. Magnification, × 200. **b** Immunofluorescence staining showed the expression of nephrin (Red) in podocyte after the indicated treatment. Magnification, × 400. Fluorescence average intensities of nestin and nephrin in podocytes after the indicated treatment. **c** Annexin V FITC staining (Green) in podocyte after the indicated treatment. Magnification, × 200. **d** Western blotting for nestin, nephrin, Bax and p53 after the indicated treatment. The data are presented as mean ± SEM. #P < 0.05 vs. control group, *P < 0.05 vs. Ang II group. $P <  0.05 vs. Ang II + RP group. (Color figure online)

To further explore the effect of nestin on the protection from rapamycin, podocytes were cultured and differentiated as previously described. We employed siRNA interference to assess the effect of nestin depletion prior to subjecting podocytes to Ang II stimulation. Nestin expression was decreased in podocytes under Ang II conditions, whereas rapamycin treatment upregulated nestin expression. Following transfection with nestin siRNA, we observed that the morphology of podocytes changed significantly, and that podocyte mutation was short or disappeared by immunofluorescence (Fig. 3a). In addition, Ang II treatment led to a decrease in nephrin expression, and a higher nephrin protein level was detected in the Ang II + RP group compared with in the Ang II group. Meanwhile, nephrin expression was downregulated when nestin-siRNA was used (Fig. 3b). The protein expression of nestin and nephrin was also detected by western blotting, and the results were consistent with the changes observed in immunofluorescence (Fig. 3d). To determine the effect of nestin on podocyte apoptosis, the cells were stained with FITC-Annexin V. Podocyte apoptosis increased significantly when treated with Ang II, as evidenced by the number of Annexin V-positive cells (green), and RP treatment reduced podocyte apoptosis. We found that the number of apoptotic podocytes was enhanced in cells co-treated with nestin siRNA compared with that in the Ang II + RP group (Fig. 3c). We also investigated the expression of Bax and p53. Western blotting analysis revealed that Bax and p53 protein levels were increased under Ang II stimulation and decreased when co-treated with Ang II and RP compared to those in the Ang II alone treatment. Corresponding to cellular apoptosis, the expression of Bax and p53 was higher when knocked down with nestin siRNA than in the Ang II + RP group (Fig. 3d).

### Nestin combines with p-mTOR, and inhibition of nestin enhances mTOR/P70S6K activation

**Fig. 4:**
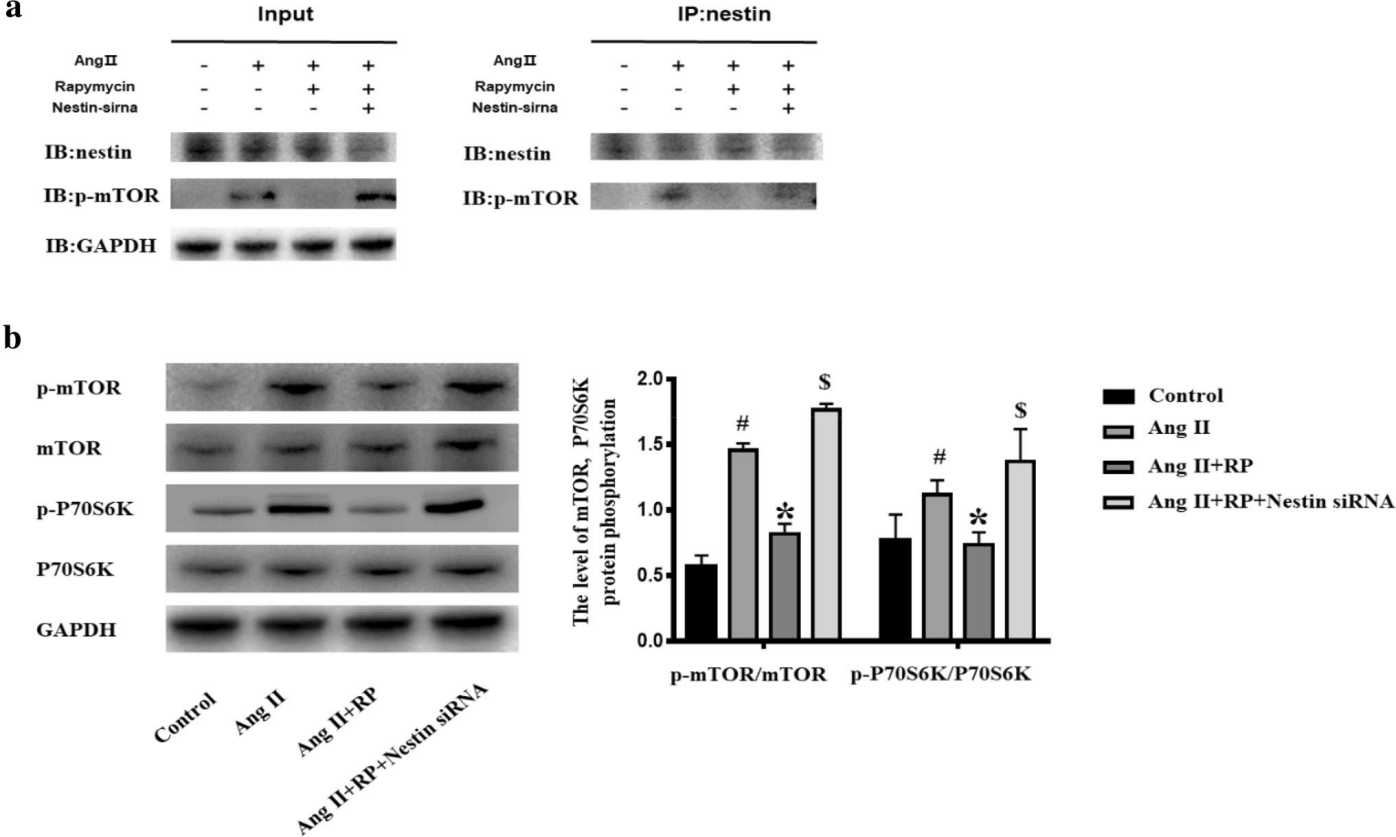
Nestin combines with p-mTOR and inhibition of nestin enhances mTOR/P70S6K activation. **a** Co-immunoprecipitation of nestin and p-mTOR in podocytes. Cell lysate was then extracted for co-immunoprecipitation with anti-nestin followed by probing with anti-nestin and anti-p-mTOR after the indicated treatment. **b** Western blotting for mTOR, p-mTOR, P70S6K, and p-P70S6K after the indicated treatment. The data are presented as mean ± SEM. ^#^P < 0.05 vs. control group, *P < 0.05 vs. Ang II group. $P < 0.05 vs. Ang II + RP group

Immunofluorescence staining showed that nestin and p-mTOR co-localized in the glomeruli. Co-IP assays were carried out to verify the interaction between nestin and p-mTOR. Co-IP demonstrated that nestin interacted with p-mTOR (Fig. 4a). We then detected the phosphorylation levels of mTOR and P70S6K proteins by western blotting (Fig. 4b), and analyzed the relative levels of mTOR, p-mTOR, P70S6K, and p-P70S6K. The levels of mTOR and P70S6K proteins were approximately equal in each group. Western blotting also revealed a significant increase in the p-mTOR and p-P70S6K protein levels in the Ang II group compared with those in the control group. Furthermore, the levels of p-mTOR and p-P70S6K proteins decreased when treated with rapamycin. Interestingly, compared with that in the Ang II + RP group, mTOR and P70S6K phosphorylation was upregulated when nestin was knocked down.

## Discussion

Several clinical and experimental studies have suggested that podocyte injury is a frequent pathological phenomenon due to various stresses and pathological stimuli, causing proteinuria formation and detachment from the glomerular basement membrane in kidney diseases [1]. Previous studies have confirmed that Ang II, an important active effector in RAS, plays a critical role in CKD progression and podocyte injury [18]. It has been reported that Ang II infusion contributes to proteinuria and hypertension in vivo, subsequently leading to podocyte injury, especially apoptosis [19]. We found that Ang II infusion induced podocyte injury in vivo, followed by a decrease in nephrin expression and loss of podocytes, as revealed in a previous study.

The mTOR pathway, which plays a central role in cellular growth, metabolism, apoptosis, and proliferation, is essential for kidney development during physiological body growth [20]. A growing number of studies have shown that the mTOR signaling pathway is an important regulator of podocyte homeostasis and tubelar transportation in renal diseases [21, 22]. In podocytes, abnormally integrated mTOR signals can be activated by numerous growth factors and cytokines. Excessive activity of mTOR complexes can result in severe pathologic effects, including mislocalization of slit diaphragm proteins, thickening of the glomerular basement membrane, loss of podocytes, and effacement of podocyte foot-press [23]. Rapamycin, an inhibitor of mTOR signaling, has been proven to be an effective therapeutic approach in animal models of glomerular disease and in clinical studies [24]. Our data showed that mTOR signaling was activated after stimulation with Ang II in vivo, and podocyte damage was involved in the activation of the mTOR pathway. In addition, we found that rapamycin attenuated podocyte apoptosis in Ang II-induced podocyte injury.

Nestin, a cytoskeleton-associated class VI intermediate filament (IF) protein, is transiently expressed in glomerular endothelial cells and tubular epithelial cells during renal development. In addition, nestin is highly expressed in mature glomerular podocytes [25]. Recent findings indicate that cytoskeleton disruption is related to podocyte injury [26]. It has previously been demonstrated that nestin plays an important role in maintaining the stability of podocytes and normal podocyte function [14]. Further, nestin expression in podocytes is closely related to proteinuria in kidney diseases, and alterations of nestin may occur to enable podocytes to undergo morphological changes [27]. In the present study, decreased nestin expression and increased p-mTOR expression were observed in Ang II-induced glomeruli. In addition, we found that rapamycin inhibited mTOR activation, followed by upregulation of nestin expression. We surmised that nestin plays an important role in the protection of rapamycin in Ang II-induced podocyte injury. To investigate the role of nestin in this process, we employed siRNA interference to assess the effect of nestin depletion prior to podocyte treatment. Our results showed that the restorative effect of rapamycin on nephrin expression was attenuated by nestin inhibition in Ang II-induced podocyte injury. The number of apoptotic podocytes increased when infected with nestin-siRNA. These results suggest that nestin is not only a marker of podocyte injury, but also a damage-promoting factor in the process.

However, the molecular mechanisms underlying this cytoprotective role remain unclear. For further study, we investigated the relationship between mTOR and nestin. Co-IP of the two proteins in podocytes verified the authenticity of the interaction between the two. Our study revealed that mTOR interacts with nestin. We then determined the phosphorylation levels of mTOR and P70S6K proteins. We found that nestin affected the protective effect of rapamycin via the mTOR/P70S6K signaling pathway. mTOR is the catalytic subunit of two distinct protein complexes, mTORC1 and mTORC2, which can be distinguished by their unique composition and different substrates [28]. The main function of mTORC1 is the activation of anabolic processes, whereas mTORC2 plays a major role in cytoskeleton organization and cell survival [29]. mTORC1 promotes protein synthesis largely through phosphorylation of p70S6 kinase1 (P70S6K) effectors. The activation of macroautophagy is a consequence of rapamycin inhibition of mTORC1. Previous findings have indicated that rapamycin disrupts the mTOR–autophagy balance by suppressing the phosphorylation of 4EBP1 and P70S6K in podocytes, resulting in the restoration of podocyte damage [30]. mTORC2 plays an important role in the regulation of cytoskeleton structure [31]. These two mTOR complexes with different functions may cross-talk with each other to balance their signaling. Moreover, either S6K1 or Akt has also been shown to control mTORC2 activity through a recently discovered negative-feedback loop [32]. It was found that mTOR/P70S6K interacts with Rac1 to recombine with the actin cytoskeleton [33]. However, the underlying mechanism remains unclear and requires further investigation.

In conclusion, our study demonstrates that rapamycin can alleviate Ang II-induced podocyte injury by upregulating the expression of nestin, both in vivo and in vitro*.* Nestin was found to play a crucial role in podocytes by modulating the mTOR/P70S6K signaling pathway through RNA interference technology and other experiments. This finding not only helps us to elucidate the mechanism of podocyte injury and the protection of rapamycin, but also provides a new potential therapeutic strategy for the treatment of CKD.

## Data Availability

The datasets analyzed during the present study are available from the corresponding author on reasonable request.
